# Synthesis of modified cyclic and acyclic dextrins and comparison of their complexation ability

**DOI:** 10.3762/bjoc.10.301

**Published:** 2014-12-02

**Authors:** Kata Tuza, László Jicsinszky, Tamás Sohajda, István Puskás, Éva Fenyvesi

**Affiliations:** 1 CycloLab Cyclodextrin R&D Laboratory Ltd, Illatos út 7, Budapest, 1097, Hungary; 2 Dipartimento di Scienza e Tecnologia del Farmaco, Universitá di Torino, via P. Giuria 9, Turin, 10125, Italy

**Keywords:** acyclodextrin, cyclodextrin, ibuprofen, maltodextrin, maltoheptaose, maltohexaose, maltooctaose

## Abstract

We compared the complex forming ability of α-, β- and γ-cyclodextrins (α-CD, β-CD and γ-CD) with their open ring analogs. In addition to the native cyclodextrins also modified cyclodextrins and the corresponding maltooligomers, functionalized with neutral 2-hydroxypropyl moieties, were synthesized. A new synthetic route was worked out via bromination, benzylation, deacetylation and debenzylation to obtain the 2-hydroxypropyl maltooligomer counterparts. The complexation properties of non-modified and modified cyclic and acyclic dextrins were studied and compared by photon correlation spectroscopy (PCS) and capillary electrophoresis (CE) using model guest compounds. In some cases cyclodextrins and their open-ring analogs (acyclodextrins) show similar complexation abilities, while with other guests considerably different behavior was observed depending on the molecular dimensions and chemical characteristics of the guests. This was explained by the enhanced flexibility of the non-closed rings. Even the signs of enantiorecognition were observed for the chloropheniramine/hydroxypropyl maltohexaose system. Further studies are planned to help the deeper understanding of the interactions.

## Introduction

The complexing ability of amylose to iodine and fatty acids is well known in the literature [1–3]. The question is raised whether the maltooligomers containing 6, 7, or 8 glucose units (G6, G7 and G8), derived from the three native α-, β-, and γ-cyclodextrins, respectively, are also suitable complexing agents. These maltooligomers are often considered as linear dextrins unable to form inclusion complexes [4]. Komiyama et al. found that there is complex formation but the complex forming ability of closed ring CDs is 2–3 orders of magnitudes higher than that of the non-cyclic, open-chain analogs [5]. Both maltohexaose and maltoheptaose (G6 and G7) caused spectral changes for guests like *p*-cresol and methyl orange in the UV–vis region. They also found that using a CPK molecular model both G6 and G7 form a favorably cyclic conformation and this explains the weak complex formation contrary to the smaller-sized non-cyclic analogs (G3, G4 and G5) not showing any interaction. In another study of Bettinetti et al. G7 was found to wrap up naproxen, taking on a cyclic conformation and forming a ‘pseudo’ inclusion complex of lower binding constant than with β-CD [6]. The helical conformations may act as ‘semicavities’ as proposed by Schurig’s group [7] who found even enantioseparation with the acetylated/silylated G6 and G7 as chiral selectors in gas chromatography. In some cases these acyclodextrins showed similar or even better enantioresolution compared to their cyclic counterparts commonly utilized in chiral separations [8].

One of the most effective techniques to explore if there are interactions between guests and hosts – particularly in the case of ionizable guest molecules – is capillary electrophoresis. The change in the mobility of a guest in the electric field in the presence of a host indicates the interaction [9–11].

Another evidence for molecular interaction can be the diminished aggregation of the guest drug; therefore photon correlation spectroscopy (PCS) is a potent tool to study the aggregation of guest molecules in the presence of the hosts [12].

In this work first the interaction of some model drugs was studied in the presence of the native cyclodextrins and the corresponding acyclodextrins. Also, modified cyclodextrins and the corresponding maltooligomers functionalized with 2-hydroxypropyl (HP) moieties, that are HP-α-CD, HP-β-CD, HP-γ-CD, HP-G6, HP-G7 and HP-G8 were synthesized and investigated as chiral additives for the separation of model compounds by CE.

## Results and Discussion

We compared the complexing ability of the three native cyclodextrins and their acyclic analogs with CE by calculating their apparent 1:1 binding constants (*K*_app_) [13]. The results are listed in Table 1.

**Table 1:** *K*_app_ values of different dextrin-drug systems (25 °C).^a^

Guest drug		α-CD	G6	β-CD	G7	γ-CD	G8

2-Phenylethylamine	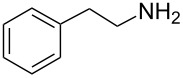	<5	<5	520	<5	300	<5
Ibuprofen	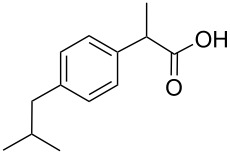	135	290	2300	200	320	280
Vinpocetine	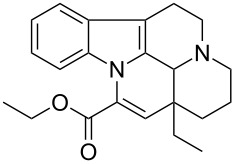	<5	<5	<5	<5	240	<5
Mefloquine	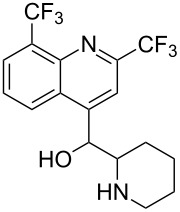	<5	<5	85	<5	15	<5
Nateglinide	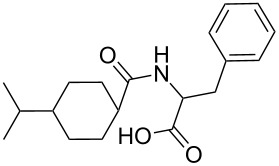	300	1400	620	300	<5	10

^a^*K*_app_ values were calculated with x-reciprocal method [13], results are displayed in M^−1^ units.

While some of the model compounds showed no interaction with most hosts studied (like vinpocetine or others not displayed), and some of them complexed by CDs only (2-phenylethylamine, mefloquine), two of the tested drugs, ibuprofen and nateglinide showed significant interaction with both the cyclic and acyclic analogs. While the apparent binding constant of the ibuprofen/β-CD system is the highest, all the acyclic derivatives showed significant affinity towards this guest irrespective of the number of glucopyranose units. Unlike CDs, the number of the glucose units might not be of primary importance in the case of ibuprofen/acyclodextrin interactions. An even more surprising finding was observed for nateglinide, as the highest interaction affinity was determined with the G6 acyclic dextrin, followed by β-CD, G7 and α-CD. In general, it can be concluded that maltooligomers have to be considered as potential complexation and interacting agents in the future that may substitute CDs in certain systems.

**Figure 1:**
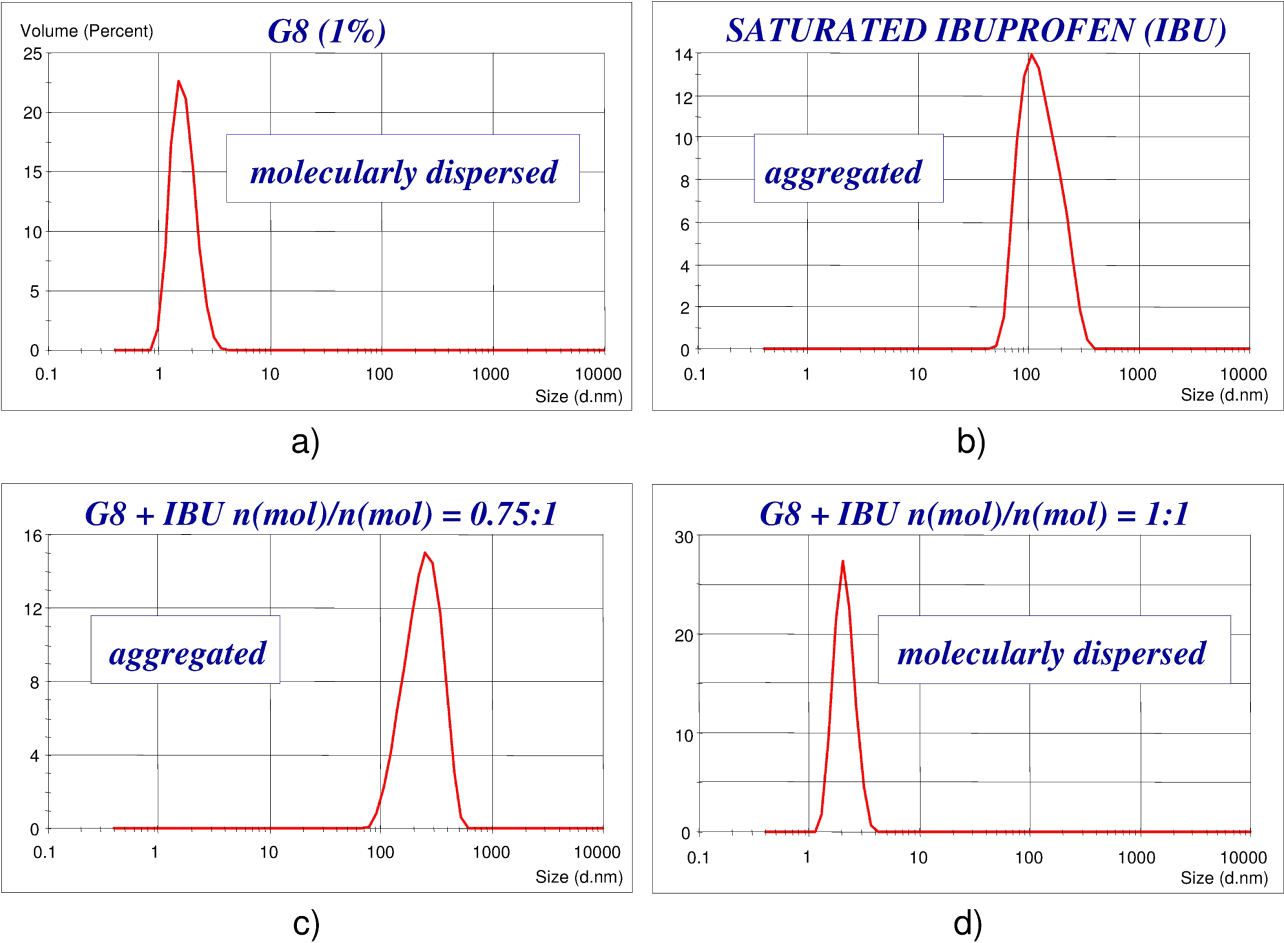
Aggregate size analysis of aqueous solutions of G8 and ibuprofen by PCS. a) G8 in 1% solution; b) saturated ibuprofen solution; c) combining ibuprofen and G8 in substoichiometric ratio (1:0.75); d) combining ibuprofen and G8 in stoichiometric ratio (1:1).

Systematic aggregation studies were performed to corroborate the existence of molecular interactions between G8 and ibuprofen as an example (Figure 1) using PCS technique. The size distribution function of G8 in 1% solution (Figure 1a) shows that G8 does not form aggregates in itself in distilled water, since the peak found at around 1.5 nm well corresponds to the dimension of a single maltooligomer molecule. A saturated solution of ibuprofen in distilled water – on the other hand – yields aggregates sized in the range of 100 nm (Figure 1b). Adding a sub-stoichiometric amount of G8 to the saturated ibuprofen solution (Figure 1c), the aggregates were still present. However, by adding G8 in equimolar amount, the aggregates were no longer detectable; the assumed associate of the drug and the maltooligomer exists in molecularly dispersed state. Based on the results of the PCS analysis, it was concluded that the interaction of the drug with G8 has an explicit stoichiometry (1:1 mol/mol), which shows similarity to a typical CD-drug interaction.

**Scheme 1:**
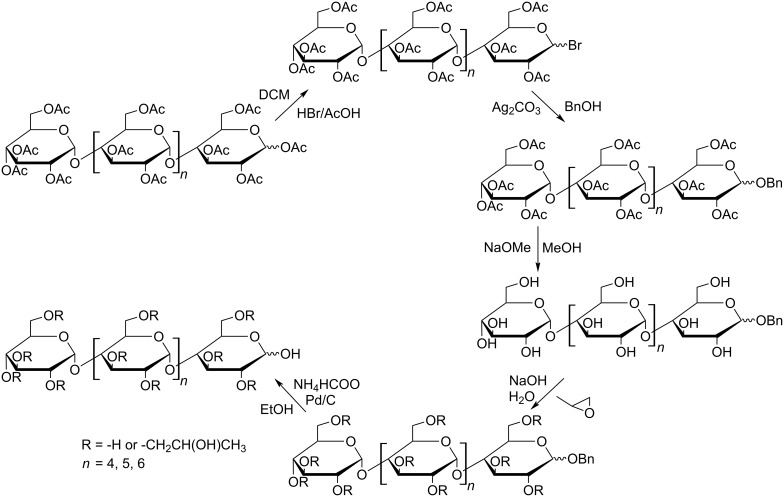
Schematic representation of the preparation of HP-substituted maltooligomers.

Based on the promising results of the preliminary complexation studies with some model drugs, modified cyclodextrins and the corresponding acyclodextrins were synthesized (Scheme 1) in order to compare their complexation behavior.

The structure elucidation of the *O*-benzyl intermediary products and the final HP-maltooligomers was performed by NMR (see Supporting Information File 1). Both proton and carbon assignments are based on the DEPT-ed-HSQC spectra.

The NMR spectra confirm the complete 1-*O*-benzylation at the glycosidic oxygen (Figures S1–S6, Supporting Information File 1). Figures S7–S12 confirm the random 2-hydroxypropylation and the lack of aromatic signals show the successful removal of the benzyl group from the target compounds. From proton spectra (Figures S7, S9, and S11, Supporting Information File 1) the degree of substitution (DS) values were calculated: 4.1, 4.3 and 4.3 for HP-G6, HP-G7, and HP-G8, respectively.

HPLC studies showed that the purity of the intermediary products was 86.5, 79.0 and 75.6% for 1-*O*-benzylmaltohexaose, 1-*O*-benzylmaltoheptaose and 1-*O*-benzylmaltooctaose, respectively. The possible impurities (maltohexaose, maltopentaose, maltotetraose, 1-*O*-benzylmaltopentaose, 1-*O*-benzylmaltotetraose, etc.) were separated. The chromatogram of 1-*O*-benzylmaltohexaose is shown as an example (Figure S13, Supporting Information File 1). The quantitative evaluation of the chromatograms are summarized in Table S3 (Supporting Information File 1).

The determination of purity of the hydroxypropyl derivatives is difficult because of the overlapping peaks yielded by products having various DS and their α- and β-anomers. Separation of the possible smaller maltooligomer impurities, like HP-maltoheptaoses, HP-maltohexaoses, HP-maltopenaoses, HP-maltotetraoses in the corresponding HP-oligomers was not successful. The chromatogram in Figure S14 (Supporting Information File 1) shows HP-maltohexaose compared to maltohexaose and *O*-benzylmaltohexaose. We assumed that the 2-hydroxypropylated derivatives have the same purity as the intermediary benzylated derivatives.

**Figure 2:**
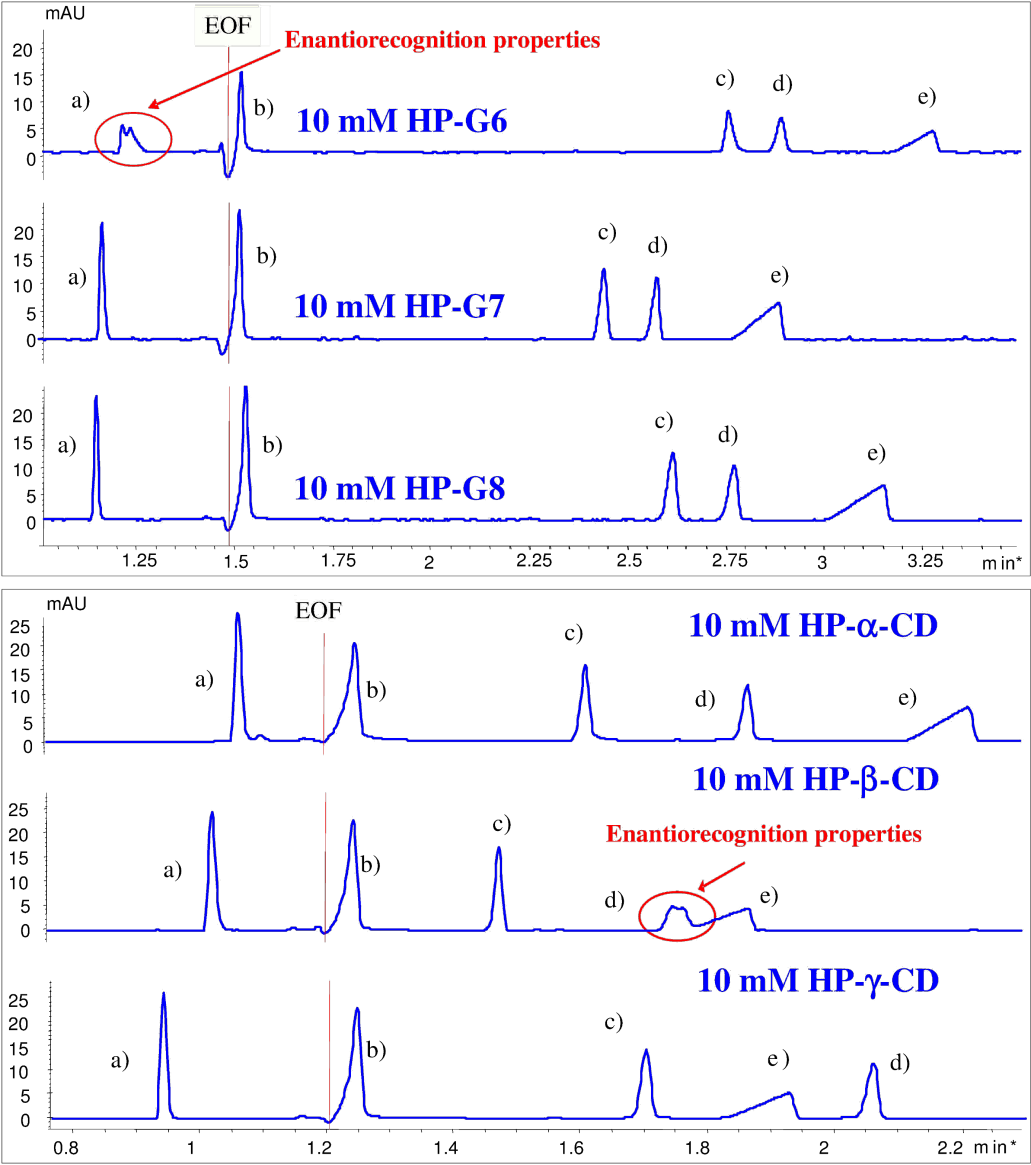
Electropherograms of tested model drugs in the presence of 2-hydroxypropylated acyclic and cyclic dextrins (assignments of the peaks are given in Table 2).

The affinity of HP-maltooligomers and the corresponding CD derivatives have been tested towards a group of guest molecules, selected based on their highly different structure and different ionic properties under these circumstances. The electrophoretic method was the same as the one applied for the native compounds. The guest candidates were chlorpheniramine, histidine, ibuprofen, camphoric acid, and chrysanthemic acid. Most of the guest compounds were racemates in order to detect enantiorecognition ability if there is any. In most cases the maltooligomers and the cyclodextrin derivatives behaved similarly, although the enantiorecognition properties frequently differed. For example, the enantiomers of chrysanthemic acid were only recognized by HP-β-CD whereas the only host resulting in a partial chiral separation of chlorpheniramine enantiomers was HP-G6 (Figure 2, fifth and first electropherogram, respectively). These preliminary results demonstrate that these derivatives have to be considered not only as possible complexation agents but also as potential chiral selectors in the future. Electropherograms of 2-hydroxypropyl derivatives and five guests are presented below (Figure 2).

Conclusions regarding the interaction affinities can be drawn based on Figure 2 and the apparent binding constants. For exploring phenomena in Figure 2 it should be noted that the closer a certain host can mobilize the analyte to the range of the electroosmotic flow (EOF, the migration range of neutral species in the system) the stronger the apparent interaction is. The mobility of the detected analytes is based on the charge-to-mass ratio. When an interaction takes place between a neutral host and a charged analyte, the mass of the associate increases while the average charge decreases. Consequently, the associate migrates closer to the EOF. As in each electropherogram the mobility of the detected peak is the average of that of the free and complexed species weighted by the molar fraction, the more pronounced this mobility change is, the stronger interaction is suggested. This indicates that in the case of chlorpheniramine (positively charged at this pH) prolongation of the migration time suggests the stronger interaction, while for the other guests (negatively charged at this pH) the contrary.

As a demonstration of the results, the association constants calculated from the electrophoretic mobilities of the five guests are presented in Table 2. As it was expected the closed ring structure is advantageous for some guest molecules resulting in 5–10 times higher association constants as compared to the proper open structures (e.g., ibuprofen with HP-α-CD/HP-G6 and HP-β-CD/HP-G7). In some cases only a slight difference was obtained (e.g., chrysantemic and camphoric acid with HP-β-CD/HP-G7) and an example was found when the open structure was superior to the closed one (chrysantemic acid with HP-γ-CD/HP-G8).

**Table 2:** *K*_app_ values of different dextrin-drug systems (25 °C).^a^

Guest drug		HP-					
		α-CD	G6	β-CD	G7	γ-CD	G8

Chlorpheniramine (a)	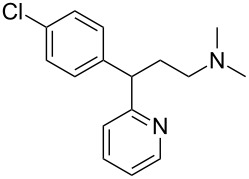	250	220	200	130	75	70
Histidine (b)	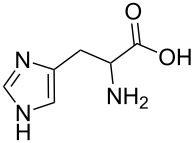	<5	<5	<5	< 5	<5	<5
Ibuprofen (c)	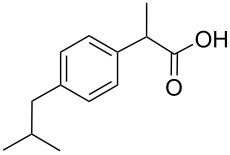	610	110	3500	450	280	210
Chrysanthemic acid (d)	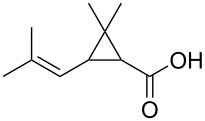	150	130	580	610	25	165
Camphoric acid (e)	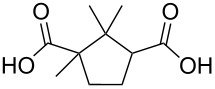	45	55	880	720	540	60

^a^*K*_app_ values were calculated with double reciprocal method [13], results are displayed in M^−1^ units.

With chlorpheniramine, CD derivatives yielded in all cases inevitably stronger interactions than the corresponding maltooligomers. It can be observed, that the hydroxypropylated CDs and the corresponding acyclodextrins possessed similar affinity towards the analyte. In the case of histidine, none of the investigated hosts showed detectable affinity. The highest stability was determined with HP-β-CD for ibuprofen and the CDs showed superiority to acyclodextrins. Similar patterns as in the case of chlorpheniramine could not be observed: the order of affinity among CDs and maltooligomers differed significantly. As for chrysanthemic acid, the strongest interaction was found with the HP-G7 maltooligomer. For this analyte the acyclodextrins showed comparable affinity for the complexation to the CD derivatives. Investigating camphoric acid the tendency of chlorpheniramine could be observed, namely that CD and the maltooligomers with the same number of glucose units had similar affinity towards the analyte except HP-γ-CD and HP-G8, the latter having much lower affinity.

## Conclusion

Non-substituted or derivatized linear dextrins can interact with small biologically active agents. In some cases this interaction was found to be stronger than or comparable to that with cyclodextrins. The enhanced affinities of the non-cyclic dextrins can be explained by their higher flexibility compared to the rigid structure of cyclodextrins. Based on the preliminary findings of this study, the acyclodextrins should be considered as potential complexation agents and chiral selectors. A deeper insight into the complexation should reveal the differences in the structures of the associates that will support the observed tendencies in interaction behavior. The synthesis and investigations of further derivatives are in progress.

## Experimental

Materials: Cyclodextrin derivatives, peracetylated maltohexa-, -hepta-, and -octaoses (>95% purity) were products of Cyclolab Ltd., Budapest, Hungary. All other reagents were purchased from Fluka, Merck and Molar Chemicals and used without further purification. All reactions were monitored by TLC. TLC: Merck 5554 Silicagel 60/F254, 5:1 v/v alcohol free chloroform/acetone for bromination, 85:15 v/v alcohol free chloroform:acetone for benzylation and 10:7 v/v dioxane:satd. aq ammonia for hydroxypropylation and debenzylation; visualization: 15% cc. H_2_SO_4_ in 96% EtOH.

### Synthesis of HP-maltooligomers

**1-Bromo-peracetyl-maltooligomers, according to** [14]: Peracetylated G6, G7 and G8 maltooligomers (3.5 mmol) were dissolved in methylene chloride (DCM, 30 mL) at 0 °C, HBr in acetic acid (105 mmol, 48% w/v) was added, and the reaction mixtures were kept at 0 °C for 4 h. The products were isolated by liquid–liquid extraction (DCM/water). Evaporation yielded 6.1/7.0/7.7 g, respectively (90–95%).

**1-*O*-Benzyl-peracetyl-maltooligomers with slight modification of** [15]: Acetobromo-G6, G7 and G8 maltooligomers (3 mmol) were dissolved in benzyl alcohol (BnOH, 120 mmol), freshly prepared dry silver carbonate (6 mmol) was added and the suspensions were stirred in the dark at room temperature for 8 h. The solids were filtered off and the products were isolated by precipitation in diisopropyl ether (DIPE). The crude products were purified by chromatography (DCM/acetone 9:1). Yield: 3.4/3.8/4.2 g (55–60%).

**1-*O*-Benzyl maltooligomers according to a modified method of** [16]: 1-*O*-Benzyl-G6, G7 and G8 maltooligomer peracetates (1.5 mmol) were dissolved in methanol (MeOH, 70 mL), sodium methoxide (4.5 mmol) was added and the solution was stirred at room temperature for 12 h. The reaction mixture was evaporated to dryness. The crude products were dissolved in water and treated with strong cation ion exchanger. The products were obtained by evaporation. Yield: 1.4/1.5/1.7 g (80–85%).

**1-*O*-Benzyl-HP (DS~4)-maltooligomers:** 1-*O*-Benzyl-G6, G7 and G8 maltooligomers (0.25 mmol) were dissolved in aqueous solutions of NaOH (2.5/1.2/1.2 mmol; 1/0.5/0.5 mL water), 1,2-propylene oxide (1.6 mmol) was added and the solutions were stirred at 5 °C for 24 h. As the TLC showed no further changes, the reaction mixtures were treated with strong ion exchanger, and then evaporated to dryness. The products were isolated by precipitation in acetone from aqueous solutions. Yield: 0.22/0.24/0.27 g (~65%).

**HP-maltooligomers according to** [17]: 1-*O*-Benzyl-HP G6, G7 and G8 -maltooligomers (0.15 mmol) were suspended in EtOH (3 mL), ammonium formiate (0.75 mmol) and 10% Pd/C (0.1 g) were added and the solutions were boiled for 30 min. Catalysts were filtered off and washed with water and the filtrates were treated with strong ion exchanger and charcoal. Evaporation of solvents yielded 0.1/0.1/0.1 g of solids, respectively (~55%).

**HP-(DS~4)-cyclodextrins:** Identical synthetic methods were used, as for the preparation of the corresponding acyclodextrin derivatives. DS = 4 was calculated based on the reactant ratios.

**CE equipment:** Agilent 7100, capillary: uncoated silica, 25 cm effective length, 20 kV voltage, 50 × 4 mbar·s hydrodynamic injection. Background electrolyte: 30 mM phosphate buffer pH set to 7.2.

**PCS Equipment**: Malvern Zetasizer Nano ZS, UK

## Supporting Information

File 1 Additional NMR and HPLC data.

## References

[1] Noltemeyer M, Saenger W (1976). Nature.

[2] Fanta G F, Shogren R L, Salch J H (1999). Carbohydr Polym.

[3] Lay Ma U V, Floros J D, Ziegler G R (2011). Carbohydr Polym.

[4] Gabelica V, Galic N, De Pauw E (2002). J Am Soc Mass Spectrom.

[5] Komiyama M, Hirai H, Kobayashi K (1986). Makromol Chem, Rapid Commun.

[6] Bettinetti G P, Mura P, Melani F, Rillosi M, Giordano F (1996). J Inclusion Phenom Mol Recognit Chem.

[7] Sicoli G, Jiang Z, Jicsinszky L, Schurig V (2005). Angew Chem.

[8] Sicoli G, Pertici F, Jiang Z, Jicsinszky L, Schurig V (2007). Chirality.

[9] Smith N W, Evans M B (1994). J Pharm Biomed Anal.

[10] Chankvetadze B, Endresz G, Blaschke G (1994). Electrophoresis.

[11] Chankvetadze B, Lindner W, Scriba G K E (2004). Anal Chem.

[12] Garnero C, Zoppi A, Genovese D, Longhi M (2010). Carbohydr Res.

[13] Rundlett K L, Armstrong D W (1996). J Chromatogr, A.

[14] Lemieux R U, Whistler R L, Wolfrom M L (1963). Tetra-O-acetyl-a-D-glucopyranosyl Bromide. Methods in Carbohydrate Chemistry.

[15] Lindberg B (1949). Acta Chem Scand.

[16] Thompson A, Wolfrom M L, Whistler R L, Wolfrom M L (1963). Deacetylation. Methods in Carbohydrate Chemistry.

[17] Jicsinszky L, Iványi R (2001). Carbohydr Polym.

