# Supplementary material for: Synthesis of modified cyclic and acyclic dextrins and comparison of their complexation ability
Source: Beilstein J Org Chem. 2014 Dec 2;10:2836–43. doi: 10.3762/bjoc.10.301 (PMC4273282; doi:10.3762/bjoc.10.301)
Supplement: File 1 — Additional NMR and HPLC data. [file Beilstein_J_Org_Chem-10-2836-s001.pdf]

Supporting Information

for

Synthesis of modified cyclic and acyclic dextrins and comparison of their complexation ability

Kata Tuza[1*], László Jicsinszky[1,2], Tamás Sohajda[1], István Puskás[1] and Éva Fenyvesi[1]

Address: [1]CycloLab Cyclodextrin R&D Laboratory Ltd, Illatos út 7, Budapest, 1097, Hungary and [2]Dipartimento di Scienza e Tecnologia del Farmaco, Universitá di Torino, via P. Giuria 9, Turin, 10125, Italy

Email: Kata Tuza -tuza@cyclolab.hu

*Corresponding author

Additional NMR and HPLC data

Characterization of the ionic derivatives by NMR

All the NMR spectra including ^1H, HSQC-DEPT (Heteronuclear Single Quantum Coherence - Distortionless Enhancement by Polarization Transfer) were recorded in D_2O on a Varian VXR-300 instrument at 300 MHz.

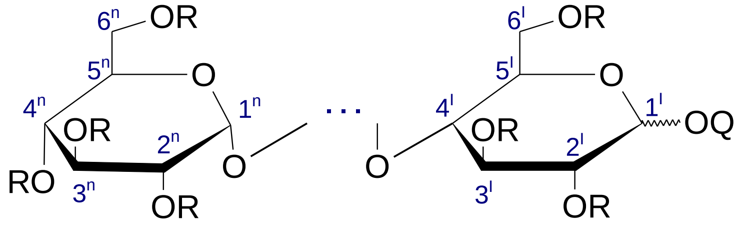

Q = Bn or H

R = H or $CH_2CH(OH)CH_3$

n = 2-8

Scheme S1: General scheme of substituted maltooligomers for NMR assignment.

Table S1: Proton assignments of 1-*O*-benzylated and (2-hydroxy)propylated maltooligosaccharides (chemical shifts are in ppm).

	1-OBn			1-OH		
	**G6**	**G7**	**G8**	**G6**	**G7**	**G8**
Benzyl, Aromatic	7.38-7.45, br m	7.42-7.47, br, m	7.40-7.60, br m	-	-	-
Benzyl, $CH_2\alpha$	4.93, d (J 11 Hz)	4.93, d (J 12 Hz)	4.93, d (J 12 Hz)	-	-	-
Benzyl, $CH_2\beta$	4.75, d (J 11 Hz)	4.76, d (J 12 Hz)	4.75, d (J 12Hz)	-	-	-
(2-Hydroxy)prop-yl CH_2	-	-	-	3.45-3.55, 3.63-68, 3.79-3.84, br	3.43-3.53, 3.61-3.66, 3.76-3.82, br	3.45-3.54, 3.62-3.66, 3.78-3.82, br
(2-Hydroxy)prop-yl CH	-	-	-	3.92-4.04, br	3.91-4.02, br	3.89-4.02, br
(2-Hydroxy)prop-yl CH_3	-	-	-	1.14, 1.16, br	1.13, 1.15, br	1.13, 1.15, br
$H1^{I}\alpha$	5.38, br	5.38, br	5.39, br	5.21, 5.52, br	5.20, 5.51, br	5.19, 5.50, br
$H1^{I}\beta$	4.52, d ($J_{1,2}$ 7.7 Hz)	4.54, d ($J_{1,2}$ 8 Hz)	4.53, d ($J_{1,2}$ 7.6 Hz)	4.63, d ($J_{1,2}$ 7.6 Hz)	4.62, d ($J_{1,2}$ 7.8 Hz)	4.62, d ($J_{1,2}$ 7.4 Hz)
$H2^{I}$	3.55, br m	3.55, br m	3.55, br m	3.55-3.72, br, unsubst<br>3.69, br, subst	3.54-3.68, br, unsubst<br>3.67, br, subst	3.54-3.68, br, unsubst<br>3.67, br, subst
$H3^{I}$	3.82, br m	3.84, br m	3.84, br m	3.76-3.87, br, unsubst<br>3.71-3.78, br, subst	3.75-3.85, br, unsubst<br>3.68-3.77, br, subst	3.74-3.86, br, unsubst<br>3.69-3.76, br, subst
$H4^{I}$	3.64-3.76, br m	3.58-3.74, br m	3.57-3.75, br m	3.44, br	3.42, br	3.43, br
$H5^{I}$	3.69-3.71, br m	3.68-3.74, br m	3.89-4.00, br m	4.03, br	4.01, br	4.01, br
$H6^{I}$	3.73-3.94, br m	3.75-3.96, br m	3.70-3.95, br m	3.74-3.93, br, unsubst<br>3.78-3.82, br, subst	3.74-3.93, br, unsubst<br>3.78-3.82, br, subst	3.74-3.93, br, unsubst<br>3.78-3.82, br, subst
$H1^{2-(n-1)}$	5.38, br d	5.38, br	5.39, br	5.39, 5.67, br	5.37, 5.65, br	5.37, 5.65, br
$H2^{2-(n-1)}$	3.60-3.63, br m	3.57-3.67, br m	3.50-3.67, br m	3.55-3.72, br, unsubst<br>3.69, br, subst	3.54-3.68, br, unsubst<br>3.67, br, subst	3.54-3.68, br, unsubst<br>3.67, br, subst

	1-OBn			1-OH		
	**G6**	**G7**	**G8**	**G6**	**G7**	**G8**
$H3^{2-(n-1)}$	3.82, br m	3.84, br m	3.84, br m	3.76-3.87, br, unsubst<br>3.71-3.78, br, subst	3.75-3.85, br, unsubst<br>3.68-3.77, br, subst	3.74-3.86, br, unsubst<br>3.69-3.76, br, subst
$H4^{2-(n-1)}$	3.64-3.76, br m	3.58-3.74, br m	3.57-3.75, br m	3.44, br	3.42, br	3.43, br
$H5^{2-(n-1)}$	3.92-3.97, br m	3.91-4.02, br m	3.62-3.73, br m	3.76-3.87, br, unsubst<br>3.71-3.78, br, subst	3.75-3.85, br, unsubst<br>3.68-3.77, br, subst	3.74-3.86, br, unsubst<br>3.69-3.76, br, subst
$H6^{2-(n-1)}$	3.73-3.94, br m	3.75-3.96, br m	3.70-3.95, br m	3.74-3.93, br, unsubst<br>3.78-3.82, br, subst	3.74-3.93, br, unsubst<br>3.78-3.82, br, subst	3.74-3.93, br, unsubst<br>3.78-3.82, br, subst
$H1^{n}$	5.38, br d	5.38, br	5.39, br	5.39, 5.67, br	5.37, 5.65, br	5.37, 5.65, br
$H2^{n}$	3.60-3.63, br m	3.57-3.67, br m	3.50-3.67, br m	3.55-3.72, br, unsubst<br>3.69, br, subst	3.54-3.68, br, unsubst<br>3.67, br, subst	3.54-3.68, br, unsubst<br>3.67, br, subst
$H3^{n}$	3.82, br m	3.84, br m	3.84, br m	3.76-3.87, br, unsubst<br>3.71-3.78, br, subst	3.75-3.85, br, unsubst<br>3.68-3.77, br, subst	3.74-3.86, br, unsubst<br>3.69-3.76, br, subst
$H4^{n}$	3.41, t ($J_{1,2}$ 9.1 Hz)	3.39, t (overlapped)	3.40, t (overlapped)	3.44, br	3.42, br	3.42, br
$H5^{n}$	3.33, t ($J_{1,2}$ 8.7 Hz)	3.35, t (overlapped)	3.33, t (overlapped)	3.27, br	3.25, br	3.25, br
$H6^{n}$	3.73-3.94, br m	3.75-3.96, br m	3.70-3.95, br m	3.74-3.93, br, unsubst<br>3.78-3.82, br, subst	3.74-3.93, br, unsubst<br>3.78-3.82, br, subst	3.74-3.93, br, unsubst<br>3.78-3.82, br, subst
1,2-Propylene-glycol CH_2	-	-	-	-	3.80, br	3.81, br
1,2-Propylene-glycol CH	-	-	-	-	3.83, br	3.84, br
1,2-Propylene-glycol CH_3	-	-	-	-	1.13, 1.15, br	1.13, 1.15, br

Table S2: Carbon assignments of 1-*O*-benzylated and (2-hydroxy)propylated maltooligosaccharides (chemical shifts are in ppm, based on DEPT-ed-HSQC experiments).

	1-OBn			1-OH		
	**G6**	**G7**	**G8**	**G6**	**G7**	**G8**
Benzyl, Aromatic	128.8, br	128.9	128.8, br	-	-	-
Benzyl, $CH_2\alpha$	71.4, 71.7	71.6, 71.7	71.5, 71.7	-	-	-
Benzyl, $CH_2\beta$	71.6	71.6	71.5, 71.7	-	-	-
(2-Hydroxy)prop-yl CH_2	-	-	-	76.1, 76.3, 76.4	76.1, 76.2, 76.4	76.1, 76.3
(2-Hydroxy)prop-yl CH	-	-	-	72.6, 73.1	73.1, 73.2	72.7, 73.1
(2-Hydroxy)prop-yl CH_3	-	-	-	18.2	18.2	18.2
$C1^{I}\alpha$	99.8, br	99.7, br	99.6-99.7, br	91.8, 98.4	91.7, 98.3	91.9, 98.3
$C1^{I}\beta$	101.2	101.2	101.2	95.6	95.7	95.8
$C2^{I}$	74.7	74.7	74.6	71.5, 72.8, unsubst<br>77.4, subst	71.5, 72.6, unsubst<br>77.2, subst	71.4, 72.6, unsubst<br>76.9, subst
$C3^{I}$	71.3	71.3	71.3	70.9 unsubst 74.7, subst	70.8 unsubst<br>74.8, subst	70.7, 71.1 unsubst<br>74.7, subst
$C4^{I}$	76.3-77.1, br	76.3-77.0, br	76.4-77.0, br	79.9	79.9	79.9
$C5^{I}$	72.9	72.9	73.4	66.3	66.6	66.1
$C6^{I}$	60.5-60.9, br	60.5-60.9, br	60.5-60.9, br	60.4, 60.6, unsubst<br>69.3, subst	60.5, unsubst<br>69.2, subst	60.4, unsubst<br>69.3, subst
$C1^{2-(n-1)}$	99.8	99.7	99.6-99.7, br	96.7, 99.6	96.7, 99.5	96.7, 99.6
$C2^{2-(n-1)}$	71.6-71.7	71.6-71.7	71.6-71.7	71.5, 72.8, unsubst<br>77.4, subst	71.5, 72.6, unsubst<br>77.2, subst	71.4, 72.6, unsubst<br>76.9, subst

	1-OBn			1-OH		
	G6	G7	G8	G6	G7	G8
$C3^{2-(n-1)}$	71.3	71.3	71.3	70.9 unsubst 74.7, subst	70.8 unsubst 74.8, subst	70.7, 71.1 unsubst 74.7, subst
$C4^{2-(n-1)}$	76.3-77.1, br	76.3-77.0, br	76.4-77.0, br	79.9	79.9	79.9
$C5^{2-(n-1)}$	73.4	73.5	73.0, 73.6	66.3	66.6	66.1
$C6^{2-(n-1)}$	60.5-60.9, br	60.5-60.9, br	60.5-60.9, br	60.4, 60.6, unsubst 69.3, subst	60.5, unsubst 69.2, subst	60.4, unsubst 69.3, subst
$C1^{n}$	99.8	99.7	99.6-99.7, br	96.7, 99.6	96.7, 99.5	96.7, 99.6
$C2^{n}$	71.6-71.7	71.6-71.7	71.6-71.7	71.5, 72.8, unsubst 77.4, subst	71.5, 72.6, unsubst 77.2, subst	71.4, 72.6, unsubst 76.9, subst
$C3^{n}$	71.3	71.3	71.3	70.9 unsubst 74.7, subst	70.8 unsubst 74.8, subst	70.7, 71.1 unsubst 74.7, subst
$C4^{n}$	69.4	69.4	69.4	69.4	69.3	69.2
$C5^{n}$	73.1	73.1	73.1	73.9	74.3	73.9
$C6^{n}$	60.5-60.9, br	60.5-60.9, br	60.5-60.9, br	60.4, 60.6, unsubst 69.3, subst	60.5, unsubst 69.2, subst	60.4, unsubst 69.3, subst
1,2-Propylene-glycol CH_2	-	-	-	-	64.5	65.6
1,2-Propylene-glycol CH	-	-	-	-	64.7	65.6
1,2-Propylene-glycol CH_3	-	-	-	-	18.2	18.2

Acquisition Time (sec)	5.4657	Comment	1_O-benzil-maltohexaoz(D1-58-A), 1H,	Date	01 Oct 2014 22:03:12
Date Stamp	01 Oct 2014 22:03:12	File Name	E:\doc\!Torino\Kata_sb_cikk\corr\beilstein\410\fid		
Frequency (MHz)	300.13	Nucleus	1H	Number of Transients	16
Origin	spect	Original Points Count	16384	Owner	guest
Points Count	65536	Pulse Sequence	zg30	Receiver Gain	181.00
SW(cyclical) (Hz)	2997.60	Solvent	DEUTERIUM OXIDE	Spectrum Offset (Hz)	1217.0212
Spectrum Type	STANDARD	Sweep Width (Hz)	2997.56	Temperature (degree C)	27.100

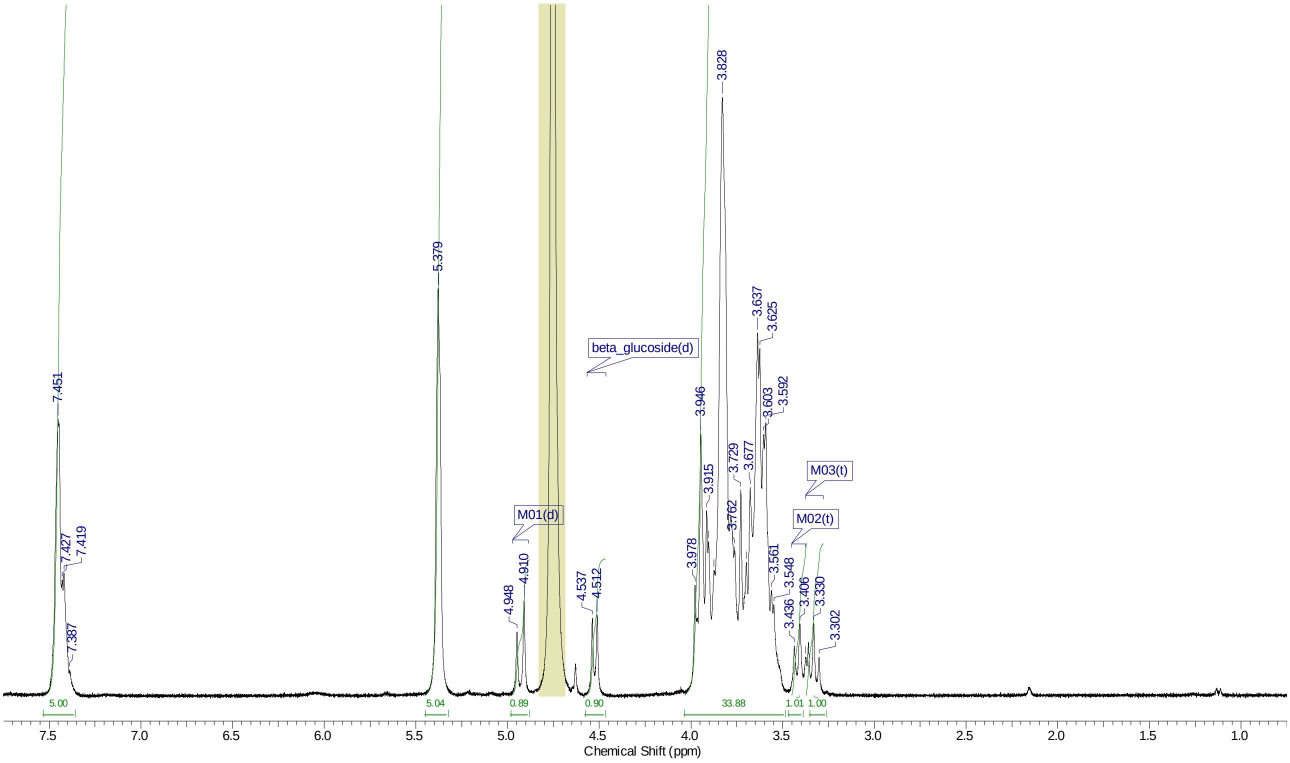

Figure S1: 300 MHz proton spectrum of 1-*O*-benzylmaltohexaose.

Acquisition Time (sec)	(0.3211, 0.0205)	Comment	5 mm QNP 1H/13C/31P/19F Z-GRD Z8352/0167	Date	09 Oct 2014 09:57:40
File Name	E:\doc\!Torino\Kata_sb_cikk\corr\beilstein\412\ser	Frequency (MHz)	(300.13, 75.47)	Nucleus	(1H, 13C)
Number of Transients	16	Origin	spect	Original Points Count	(512, 256)
Owner	guest	Points Count	(1024, 1024)	Pulse Sequence	hsqcedetgp
Solvent	D2O	Spectrum Type	HSQC-DEPT	Sweep Width (Hz)	(1594.39, 12500.00)
Temperature (degree C)	27.300	Title	1_O-benzil-maltohexaoz(D1-58-A), ed-gs-HSQC,		

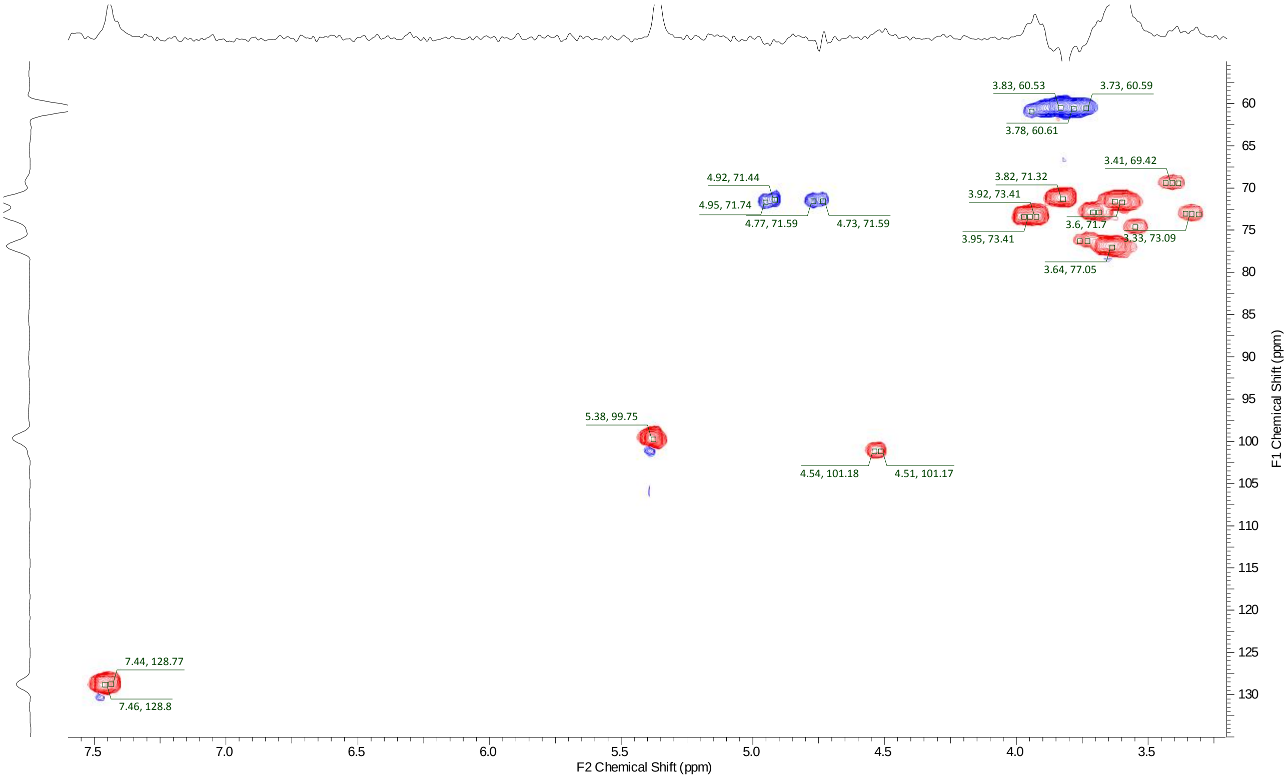

Figure S2: DEPT-ed-HSQC spectrum of 1-*O*-benzylmaltohexaose.

Acquisition Time (sec)	5.4657	***Comment***	1_O-benzil-maltoheptaoz(D1-58-B), 1H,			***Date***	02 Oct 2014 02:14:56
Date Stamp	02 Oct 2014 02:14:56			***File Name***	E:\doc\!Torino\Kata_sb_cikk\corr\beilstein\430\fid		
Frequency (MHz)	300.13	***Nucleus***	1H	***Number of Transients***	16	***Origin***	spect
Original Points Count	16384	***Owner***	guest	***Points Count***	65536	***Pulse Sequence***	zg30
Receiver Gain	181.00	***SW(cyclical) (Hz)***	2997.60	***Solvent***	DEUTERIUM OXIDE		
Spectrum Offset (Hz)	1216.0607	***Spectrum Type***	STANDARD	***Sweep Width (Hz)***	2997.56	***Temperature (degree C)***	27.400

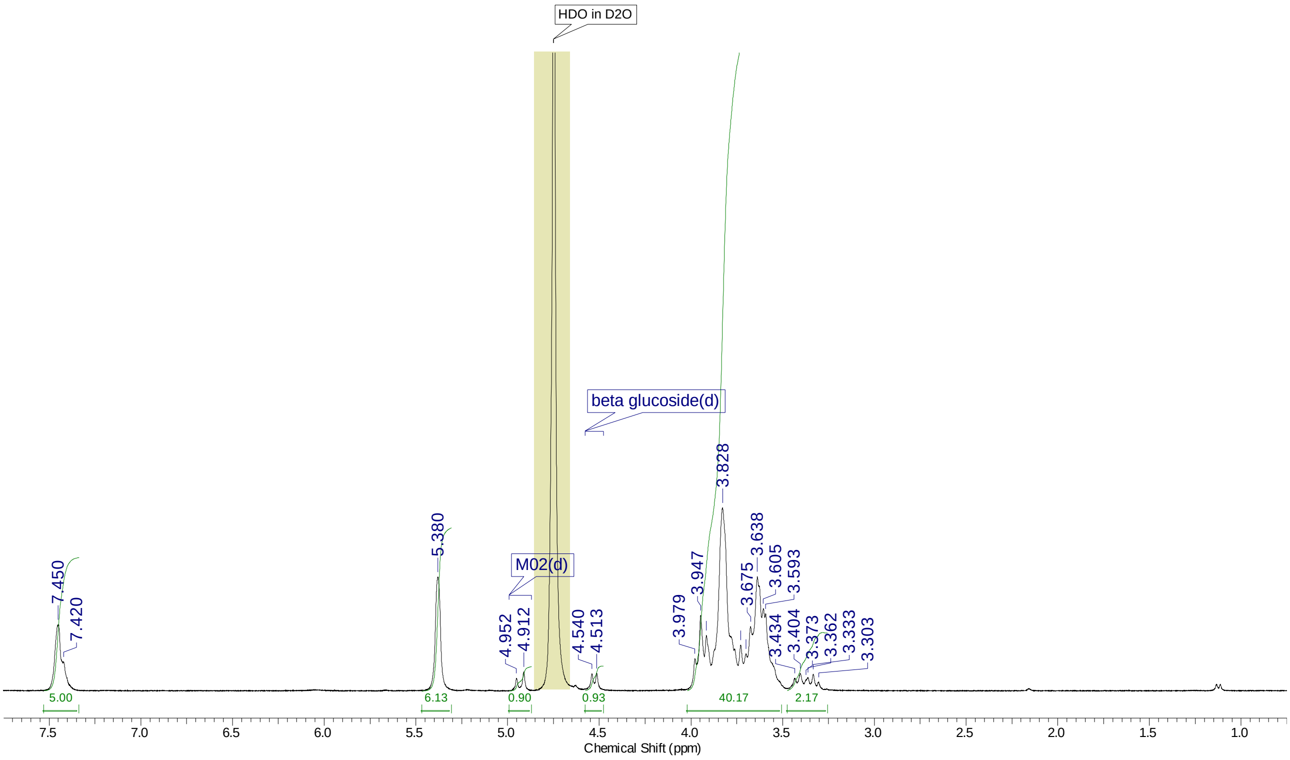

Figure S3: 300 MHz proton spectrum of 1-*O*-benzylmaltoheptaose.

Acquisition Time (sec)	(0.3244, 0.0205)	***Comment***	5 mm QNP 1H/13C/31P/19F Z-GRD Z8352/0167			***Date***	09 Oct 2014 10:12:06
File Name	E:\doc\!Torino\Kata_sb_cikk\corr\beilstein\432\ser			***Frequency (MHz)***	(300.13, 75.47)	***Nucleus***	(1H, 13C)
Number of Transients	16	***Origin***	spect	***Original Points Count***	(512, 256)	***Owner***	guest
Points Count	(1024, 1024)	***Pulse Sequence***	hsqcedetgp	***Solvent***	D2O	***Spectrum Type***	HSQC-DEPT
Sweep Width (Hz)	(1578.28, 12500.00)	***Temperature (degree C)***	27.600	***Title***	1_O-benzil-maltoheptaoz(D1-58-B), 1H,		

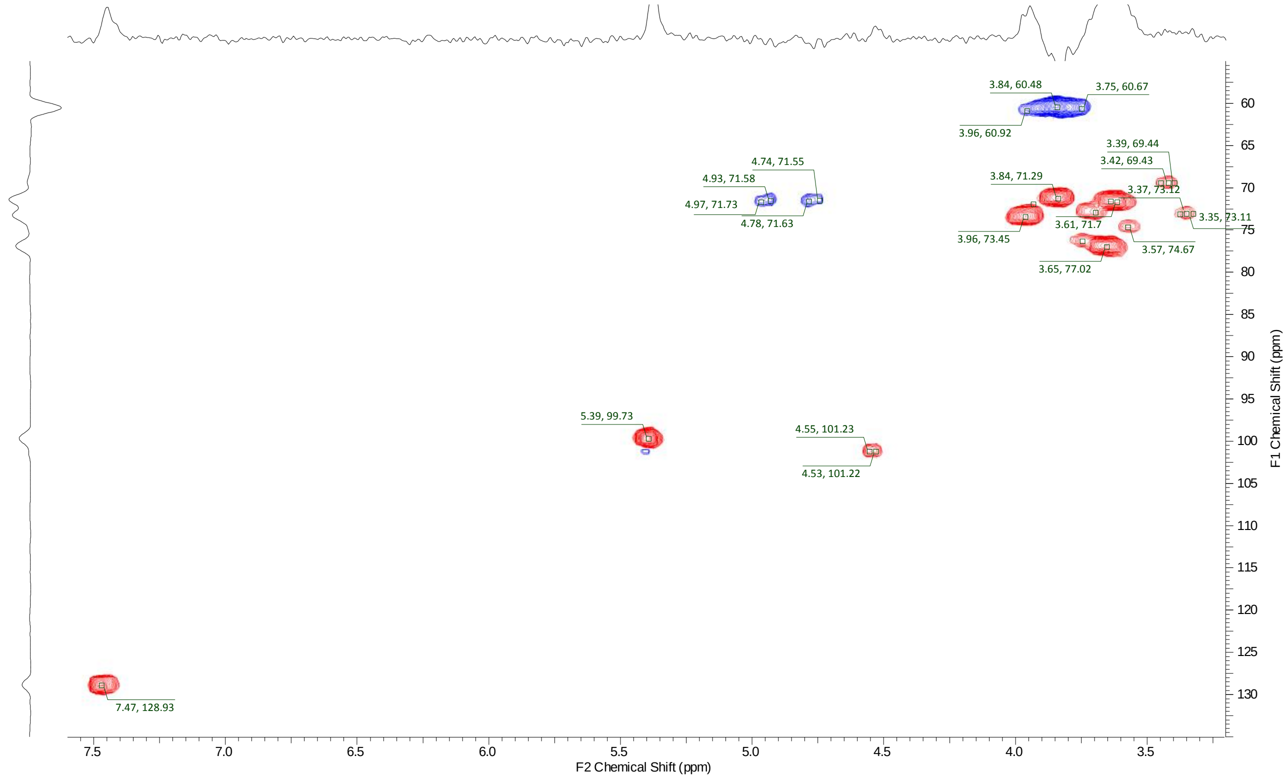

Figure S4: DEPT-ed-HSQC spectrum of 1-*O*-benzylmaltoheptaose.

Acquisition Time (sec)	5.4657	***Comment***	1_O-benzil-maltooktaoz(D1-58-C), 1H,			***Date***	02 Oct 2014 04:20:48
Date Stamp	02 Oct 2014 04:20:48			***File Name***	E:\doc\!Torino\Kata_sb_cikk\corr\beilstein\440\fid		
Frequency (MHz)	300.13	***Nucleus***	1H	***Number of Transients***	16	***Origin***	spect
Original Points Count	16384	***Owner***	guest	***Points Count***	65536	***Pulse Sequence***	zg30
Receiver Gain	181.00	***SW(cyclical) (Hz)***	2997.60	***Solvent***	DEUTERIUM OXIDE		
Spectrum Offset (Hz)	1216.7925	***Spectrum Type***	STANDARD	***Sweep Width (Hz)***	2997.56	***Temperature (degree C)***	27.500

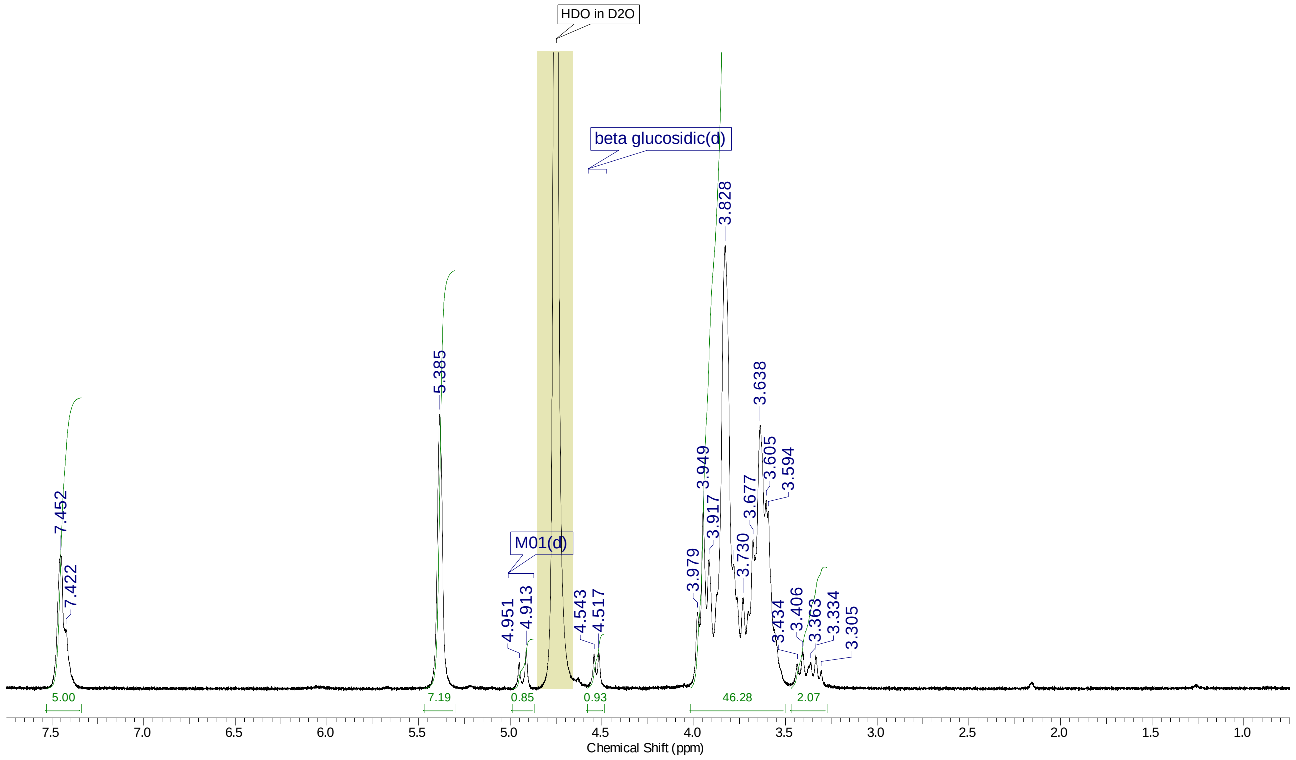

Figure S5: 300 MHz proton spectrum of 1-*O*-benzylmaltooctaose.

Acquisition Time (sec)	(0.3211, 0.0205)	***Comment***	5 mm QNP 1H/13C/31P/19F Z-GRD Z8352/0167			***Date***	09 Oct 2014 09:57:40
File Name	E:\doc\!Torino\Kata_sb_cikk\corr\beilstein\412\ser			***Frequency (MHz)***	(300.13, 75.47)	***Nucleus***	(1H, 13C)
Number of Transients	16	***Origin***	spect	***Original Points Count***	(512, 256)	***Owner***	guest
Points Count	(1024, 1024)	***Pulse Sequence***	hsqcedetgp	***Solvent***	D2O	***Spectrum Type***	HSQC-DEPT
Sweep Width (Hz)	(1594.39, 12500.00)	***Temperature (degree C)***	27.300	***Title***	1_O-benzil-maltohexaoz(D1-58-A), ed-gs-HSQC,		

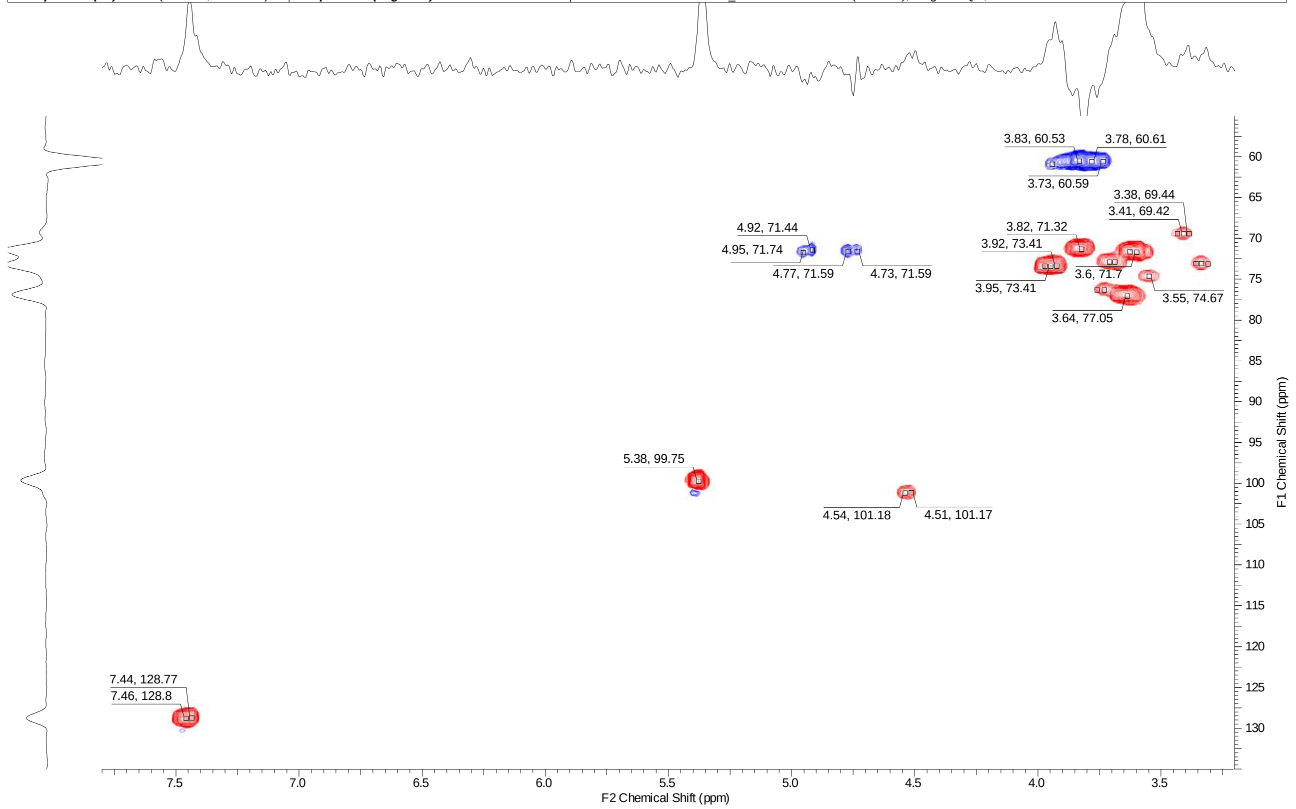

Figure S6: DEPT-ed-HSQC spectrum of 1-*O*-benzylmaltooctaose.

Acquisition Time (sec)	5.4657	***Comment***	(2hidroxi)propil-maltohexaoz(D1-68-A), 1H,			***Date***	02 Oct 2014 00:09:04		
Date Stamp	02 Oct 2014 00:09:04			***File Name***	E:\doc\!Torino\Kata_sb_cikk\corr\beilstein\420\fid			***Frequency (MHz)***	300.13
Nucleus	1H	***Number of Transients***	16	***Origin***	spect	***Original Points Count***	16384	***Owner***	guest
Points Count	65536	***Pulse Sequence***	zg30	***Receiver Gain***	181.00	***SW(cyclical) (Hz)***	2997.60	***Solvent***	DEUTERIUM OXIDE
Spectrum Offset (Hz)	1217.0212	***Spectrum Type***	STANDARD	***Sweep Width (Hz)***	2997.56	***Temperature (degree C)***	27.600		

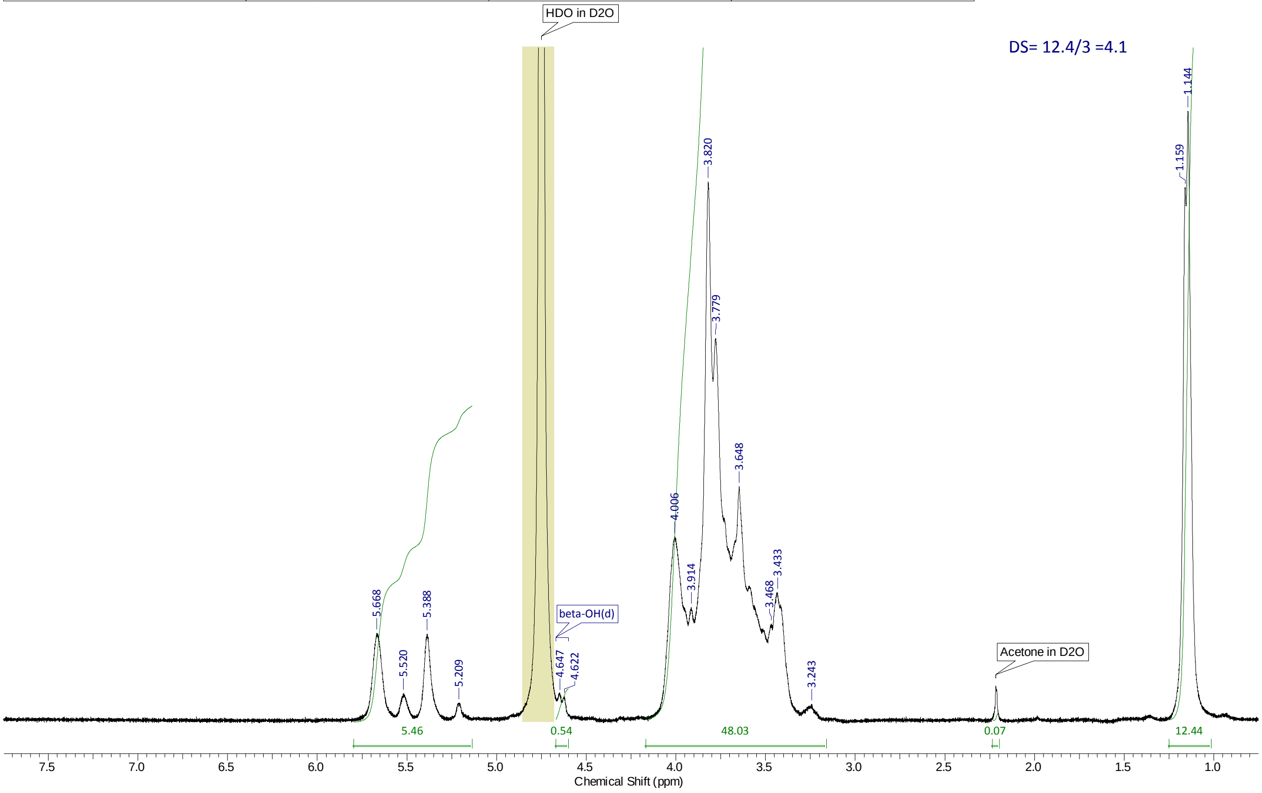

Figure S7: 300 MHz proton spectrum of (2-hydroxy)propylated maltohexaose.

Acquisition Time (sec)	(0.2851, 0.0205)	***Comment***	5 mm QNP 1H/13C/31P/19F Z-GRD Z8352/0167			***Date***	09 Oct 2014 10:37:24
File Name	E:\doc\!Torino\Kata_sb_cikk\corr\beilstein\422\ser			***Frequency (MHz)***	(300.13, 75.47)	***Nucleus***	(1H, 13C)
Number of Transients	16	***Origin***	spect	***Original Points Count***	(512, 256)	***Owner***	guest
Points Count	(2048, 1024)	***Pulse Sequence***	hsqcedetgp	***Solvent***	D2O	***Spectrum Type***	HSQC-DEPT
Sweep Width (Hz)	(1795.98, 12500.00)	***Temperature (degree C)***	27.600	***Title***	(2hidroxi)propil-maltohexaoz(D1-68-A), gs-HSQC,		

AutoPhase	Ambiguous Phase

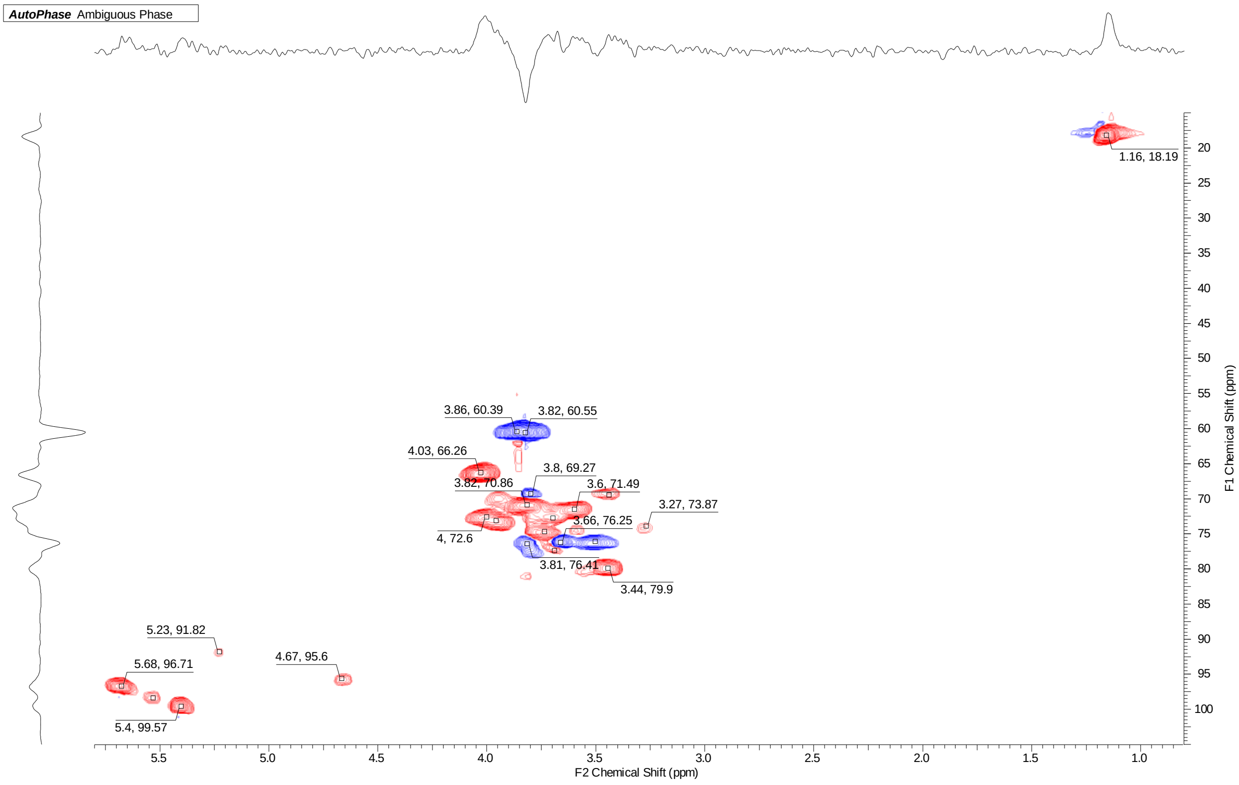

Figure S8: DEPT-ed-HSQC spectrum of (2-hydroxy)propylated maltohexaose.

**Acquisition Time (sec)**	5.4657	**Comment**	(2-hidroxi)propil-heptaoz(D1-68-B), 1H,	**Date**	02 Oct 2014 14:48:00
**Date Stamp**	02 Oct 2014 14:48:00	**File Name**	E:\doc\!Torino\Kata_sb_cikk\corr\beilstein\450\fid	**Frequency (MHz)**	300.13
**Nucleus**	1H	**Number of Transients**	16	**Origin**	spect
**Original Points Count**	16384	**Owner**	guest	**Points Count**	65536
**Pulse Sequence**	zg30	**Receiver Gain**	181.00	**SW(cyclical) (Hz)**	2997.60
**Solvent**	DEUTERIUM OXIDE	**Spectrum Offset (Hz)**	1218.1189	**Spectrum Type**	STANDARD
**Sweep Width (Hz)**	2997.56	**Temperature (degree C)**	26.100		

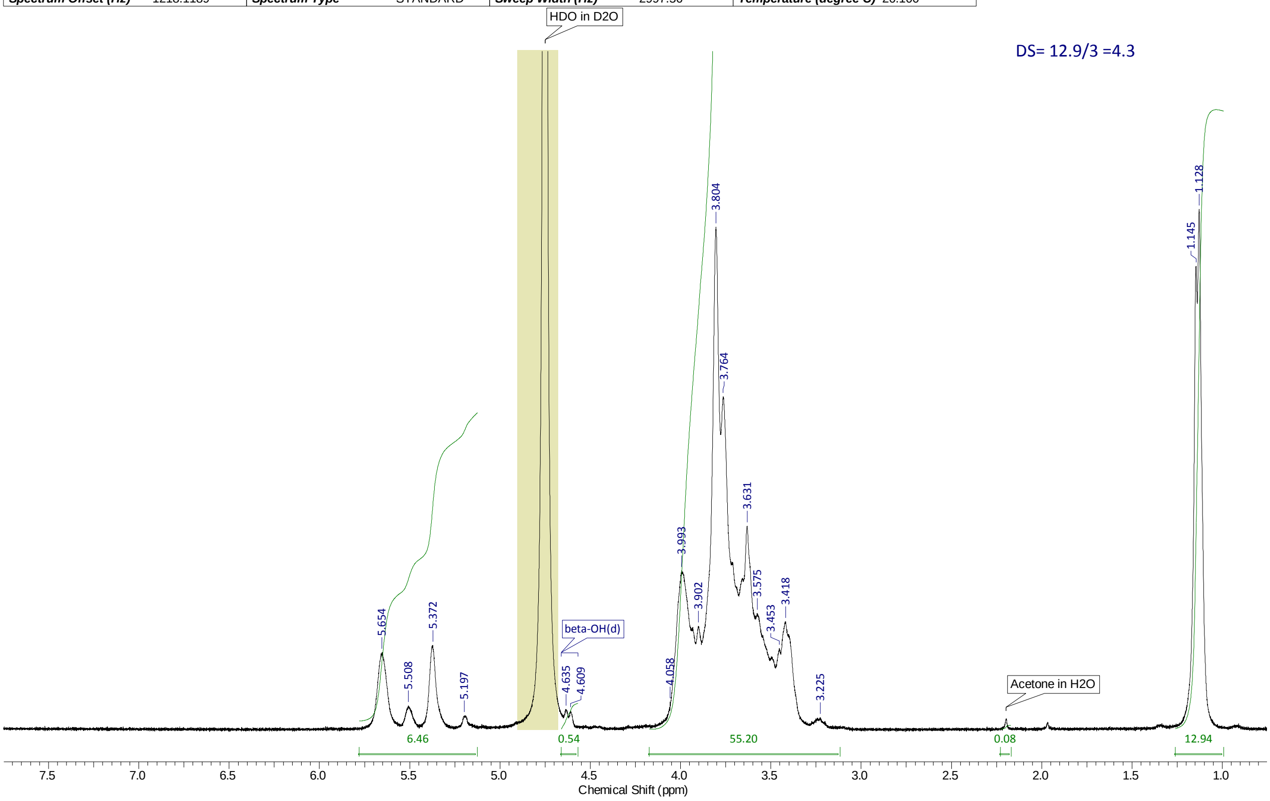

Figure S9: 300 MHz proton spectrum of (2-hydroxy)propylated maltoheptaose.

**Acquisition Time (sec)**	(0.2834, 0.0205)	**Comment**	5 mm QNP 1H/13C/31P/19F Z-GRD Z8352/0167	**Date**	08 Oct 2014 17:21:10
**File Name**	E:\doc\!Torino\Kata_sb_cikk\corr\beilstein\452\ser	**Frequency (MHz)**	(300.13, 75.47)	**Nucleus**	(1H, 13C)
**Number of Transients**	16	**Origin**	spect	**Original Points Count**	(512, 256)
**Owner**	guest	**Points Count**	(1024, 1024)	**Pulse Sequence**	hsqcedetgp
**Solvent**	D2O	**Spectrum Type**	HSQC-DEPT	**Sweep Width (Hz)**	(1806.36, 12500.00)
**Temperature (degree C)**	26.200	**Title**	(2-hidroxi)propil-heptaoz(D1-68-B), 1H,		

AutoPhase Ambiguous Phase

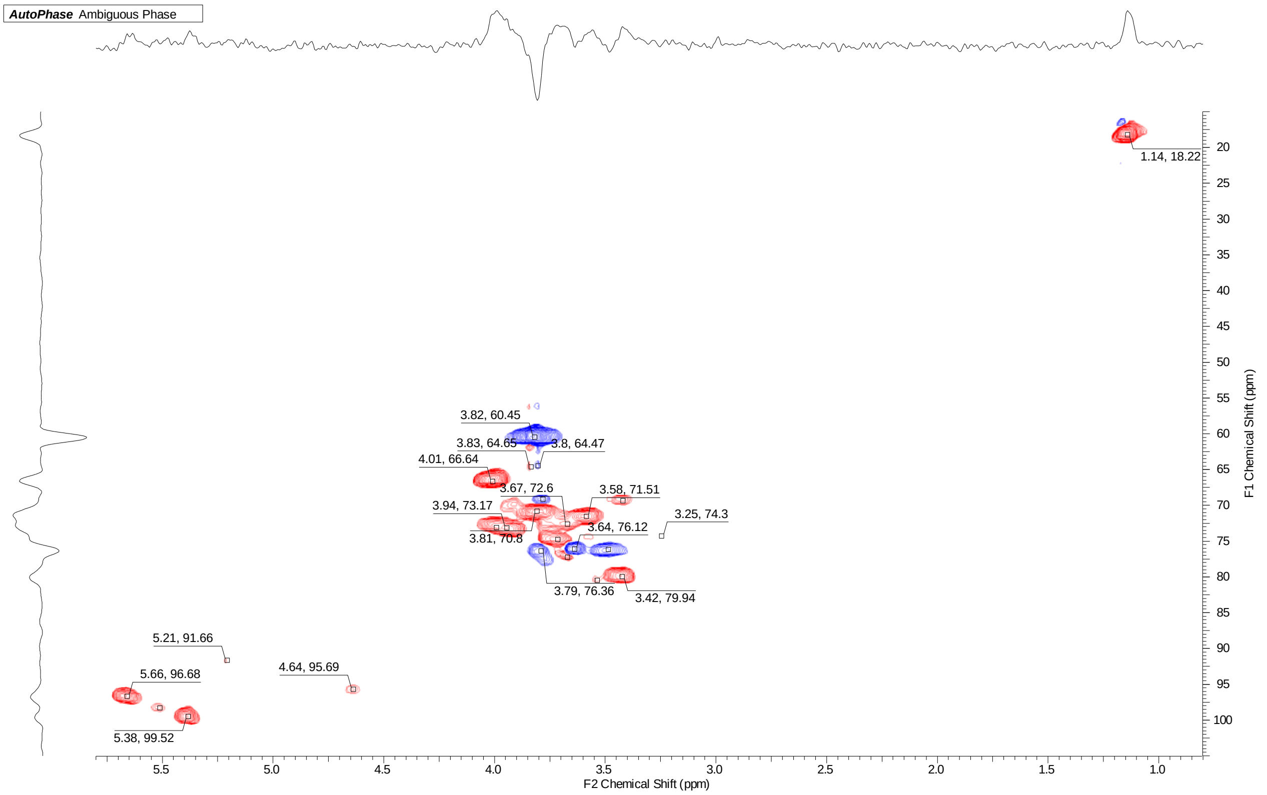

Figure S10: DEPT-ed-HSQC spectrum of (2-hydroxy)propylated maltoheptaose.

Acquisition Time (sec)	5.4657	***Comment***	(2-hidroxi)propil-oktaoz(D1-68-C), 1H,			***Date***	02 Oct 2014 16:53:52		
Date Stamp	02 Oct 2014 16:53:52			***File Name***	E:\doc\!Torino\Kata_sb_cikk\corr\beilstein\460\fid			***Frequency (MHz)***	300.13
Nucleus	1H	***Number of Transients***	16	***Origin***	spect	***Original Points Count***	16384	***Owner***	guest
Points Count	65536	***Pulse Sequence***	zg30	***Receiver Gain***	181.00	***SW(cyclical) (Hz)***	2997.60	***Solvent***	DEUTERIUM OXIDE
Spectrum Offset (Hz)	1217.6157	***Spectrum Type***	STANDARD	***Sweep Width (Hz)***	2997.56	***Temperature (degree C)***	26.100		

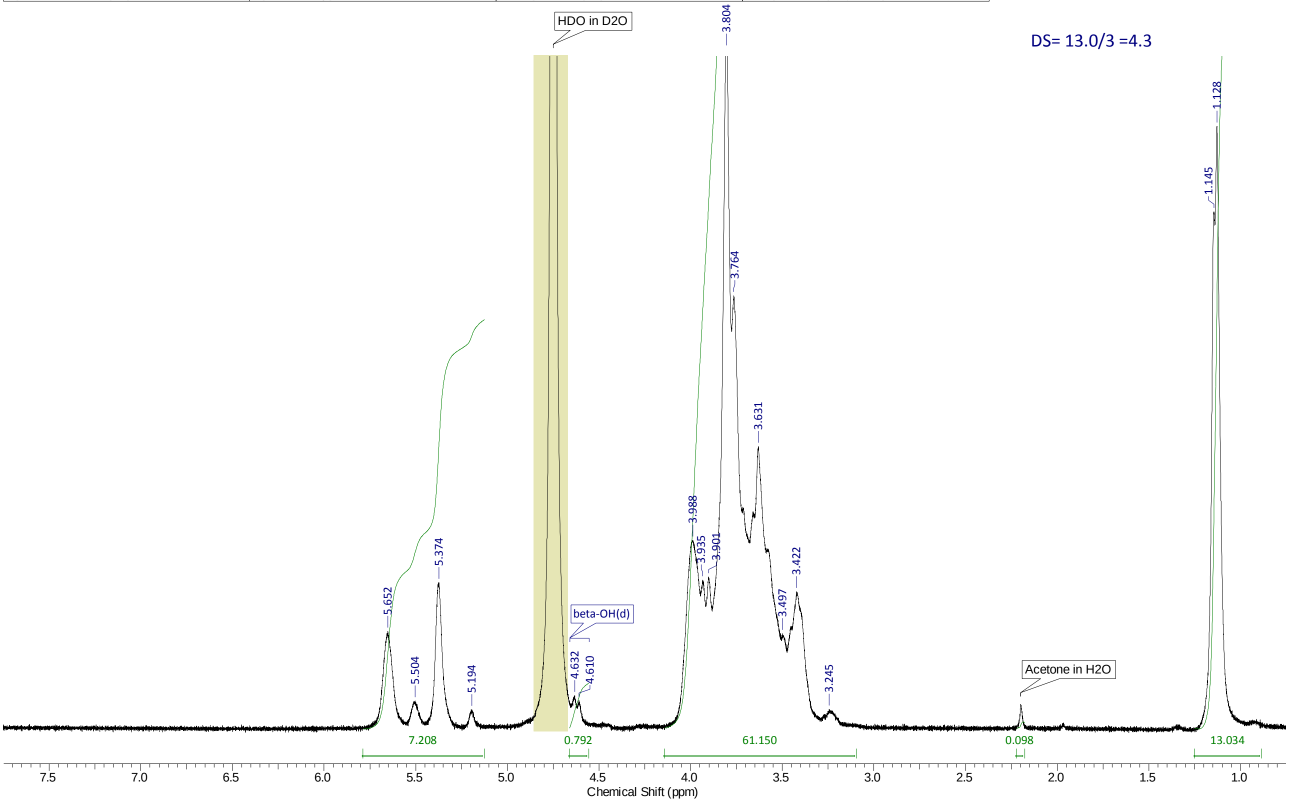

Figure S11: 300 MHz proton spectrum of (2-hydroxy)propylated maltooctaose.

Acquisition Time (sec)	(0.2834, 0.0205)	***Comment***	5 mm QNP 1H/13C/31P/19F Z-GRD Z8352/0167			***Date***	08 Oct 2014 15:48:14
File Name	E:\doc\!Torino\Kata_sb_cikk\corr\beilstein\462\ser			***Frequency (MHz)***	(300.13, 75.47)	***Nucleus***	(1H, 13C)
Number of Transients	16	***Origin***	spect	***Original Points Count***	(512, 256)	***Owner***	guest
Points Count	(1024, 1024)	***Pulse Sequence***	hsqcedetgp	***Solvent***	D2O	***Spectrum Type***	HSQC-DEPT
Sweep Width (Hz)	(1806.36, 12500.00)	***Temperature (degree C)***	26.200	***Title***	(2-hidroxi)propil-oktaoz(D1-68-C), 1H,		

AutoPhase	Ambiguous Phase

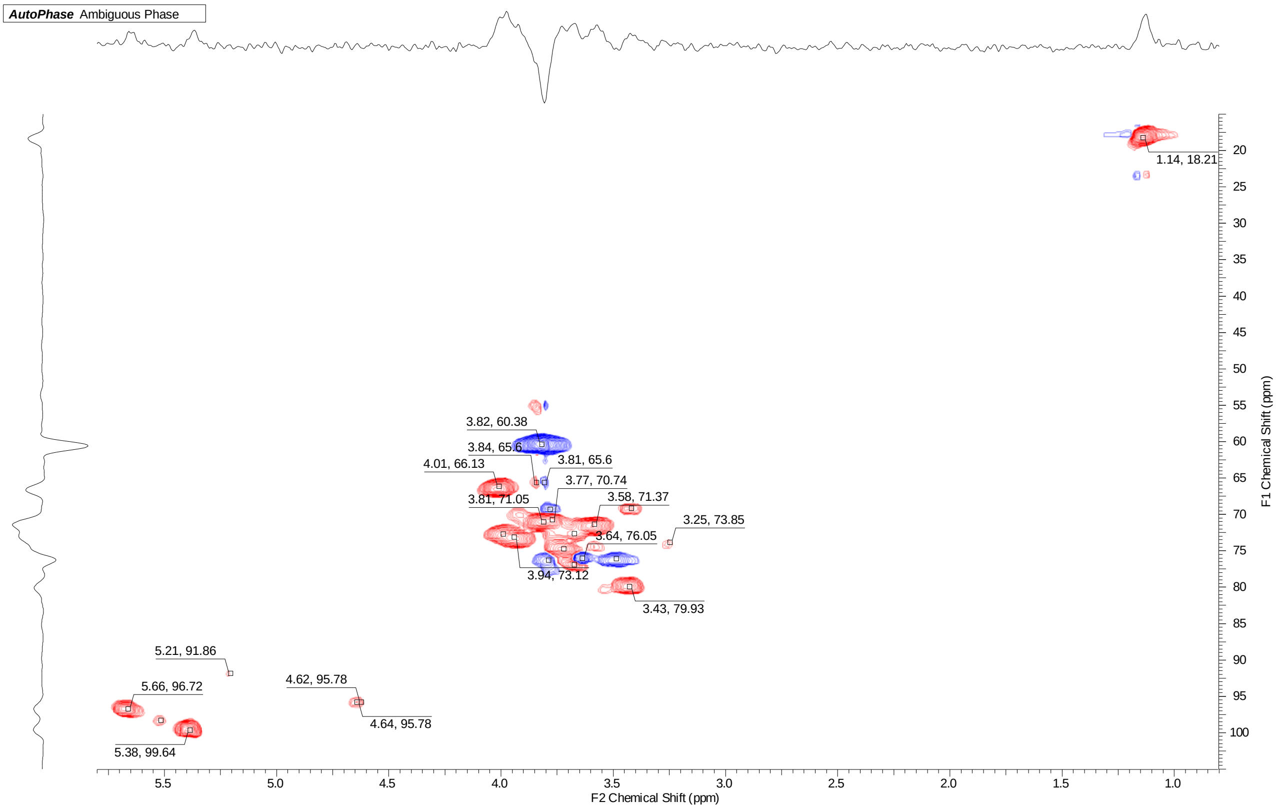

Figure S12: DEPT-ed-HSQC spectrum of (2-hydroxy)propylated maltooctaose.

Characterization of the benzylated maltooligomers with HPLC

Agilent HPLC measuring system with Refractive Index Detector and/or DAD detector was used.
The used HPLC column was Inertsil HILIC, 150 × 4.6 mm, particle size 5 μm (GL Sciences Inc.)
The most appropriate mobile phase contained acetonitrile:water = 69:31.
The flow rate was 1.0 mL/min. The column temperature was set to 30 °C; the injection volume from the sample solution (concentration: 2 mg/mL mobile phase) was 100 μL.

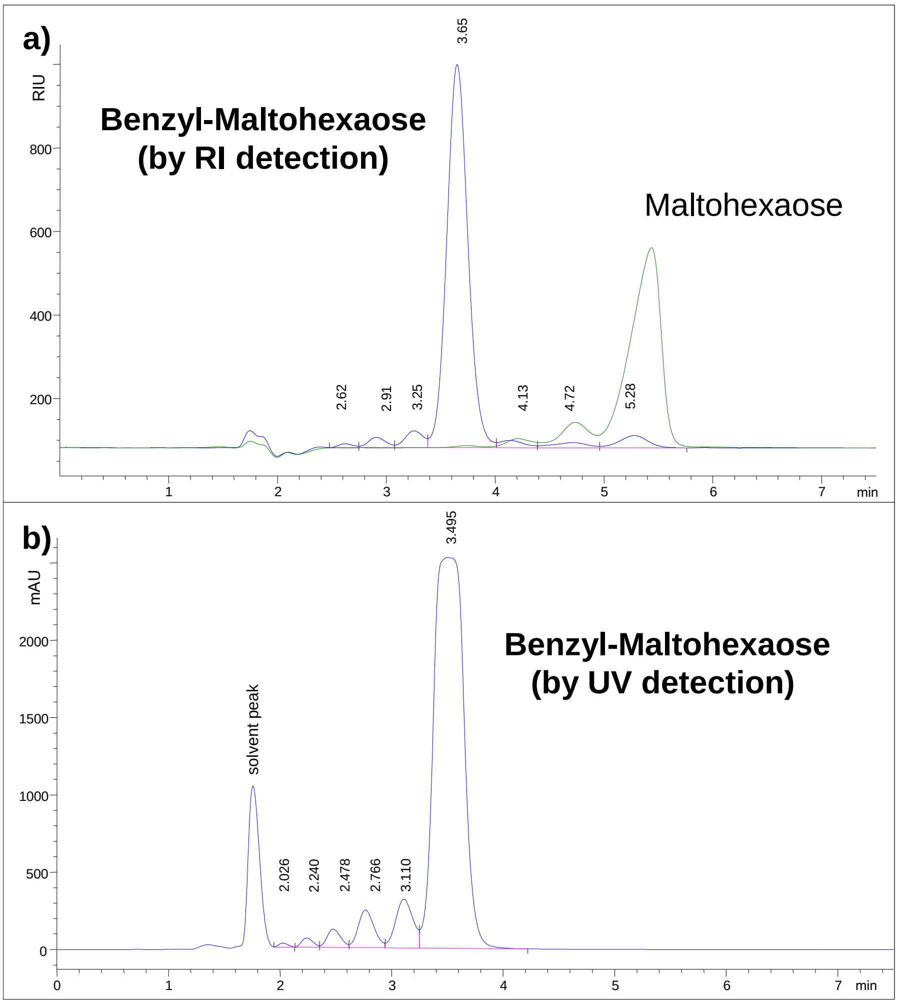

Figure S13: HPLC chromatogram of 1-*O*-benzylmaltohexaose and maltohexaose using refractive index detector (a) and DAD detector (b).

Table S3: Area percentage of the peaks detected by HPLC.

sample	Composition (in area %)								
	Retention time of peak (min.)								
	2.6	2.9	3.3	3.6	4.1	4.6	5.1/5.3	5.9	6.6
Benzyl-Maltohexaose	0.6	1.9	3.3	**86.5**	1.9	2.0	3.9		
Benzyl-Maltoheptaose	0.2	1.0	3.2	7.5	**79.0**	2.5	2.7	3.8	
Benzyl-Maltooctaose	0.4	1.1	2.6	4.8	5.5	**75.6**	2.9	2.2	5.1

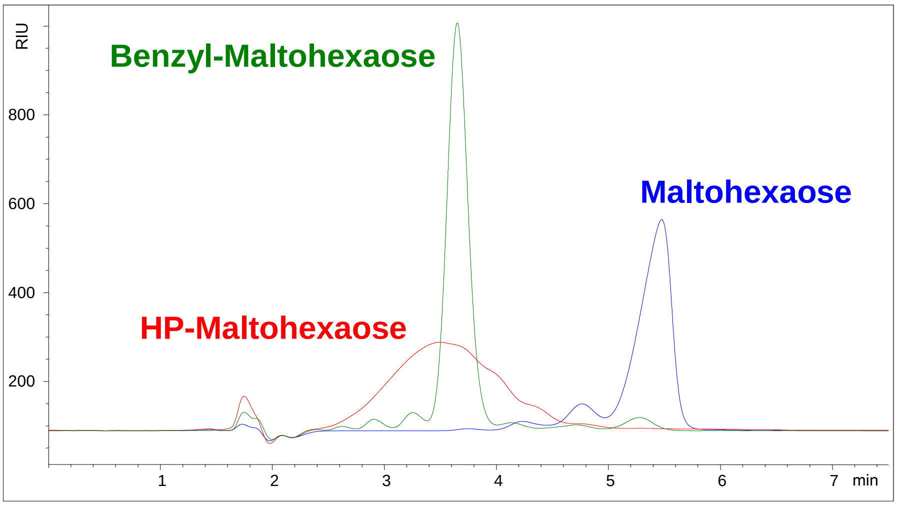

Figure S14: HPLC chromatogram of HP-maltohexaose compared to maltohexaose and O-benzyl-maltohexaose.
